# Semi-focal bone transport versus traditional bone transport technique for the management of large tibial bone defects after trauma

**DOI:** 10.1038/s41598-024-58548-z

**Published:** 2024-04-05

**Authors:** Qian Wang, Teng Ma, Zhong Li, Kun Zhang, Qiang Huang

**Affiliations:** Department of Orthopedics, Hong Hui Hospital, Xi’an Jiaotong University, Xi’an, 710054 Shaanxi China

**Keywords:** Tibial defects, Semicylindrical, Bone transport, Semi-focal, Calcium sulfate, Fracture repair, Trauma

## Abstract

How to deal with large tibial bone defects is still controversial. The purpose of this research was to compare the semi-focal bone transport (SFBT) technique with traditional bone transport (TBT) technique for treating such patients. Sixty-two patients were included and retrospectively analyzed. In all cases, after radical debridement large tibial bone defects remained. Patients were treated by the SFBT or TBT technique. The distraction, consolidation duration and complications were recorded by the patients’ medical files. Based on the Association for the Study and Application of Methods of Ilizarov (ASAMI) standard, the bone and functional results were evaluated. The mean bone defect size was 7.7 ± 1.6 cm and 7.5 ± 2.1 cm for SFBT and TBT patients. The mean external fixation index (EFI) was 1.51 ± 0.14 months/cm and 1.89 ± 0.25 months/cm for SFBT and TBT patients (*p* < 0.05), respectively. With respect to bone and function results, there was no significant differences between the two groups (*p* > 0.05). The mean number of complications per patient was 1.1 ± 0.6 and 1.6 ± 0.7 for SFBT and TBT patients (*p* < 0.05). Compared to the traditional bone transport technique, patients using the semi-focal bone transport technique achieved better clinical effects, including shorter EFI and less complications. Therefore, the SFBT technique could be a new option for patients with large tibial bone defects.

## Introduction

Large tibial bone defects are encountered commonly by trauma surgeons. Such injuries may be caused by acute trauma or infection after internal fixation. These patients may suffer from large skin and soft tissue defects, or even vascular and nerve injuries simultaneously. Management of such injuries is sometimes challenging, even for skillful and experienced orthopedic surgeons. Several reconstruction strategies have been applied and passed the test of clinical practice, ranging from autologous bone transplantation, Masquelet technique, Ilizarov bone transport technique, vascularized or non-vascularized fibular transplantation^1–6^.

Autologous bone transplantation is suitable for a small range of bone defects, usually less than five centimeters. The effect of cancellous bones is better than cortical bones, whose vitality is eight times that of cortical bones^7^. Masquelet technique was reported for the first time in 1986 and has been widely used for the management of segmental bone defects according to staged surgery principles^1,4^. Masquelet technique is essentially still a bone transplantation technique. The inducer used during surgery is polymethylmethacrylate (PMMA) bone cement, which needs to be removed by a secondary surgery^4^. However, for patients with large bone defects, the amount of autogenous bones is limited. The soft tissues around the tibia are weak. The local soft tissue conditions are usually weaker for patients suffering from acute trauma or osteomyelitis. This may lead to poor healing of implanted bones and even infection recurrence.

**Figure 1:**
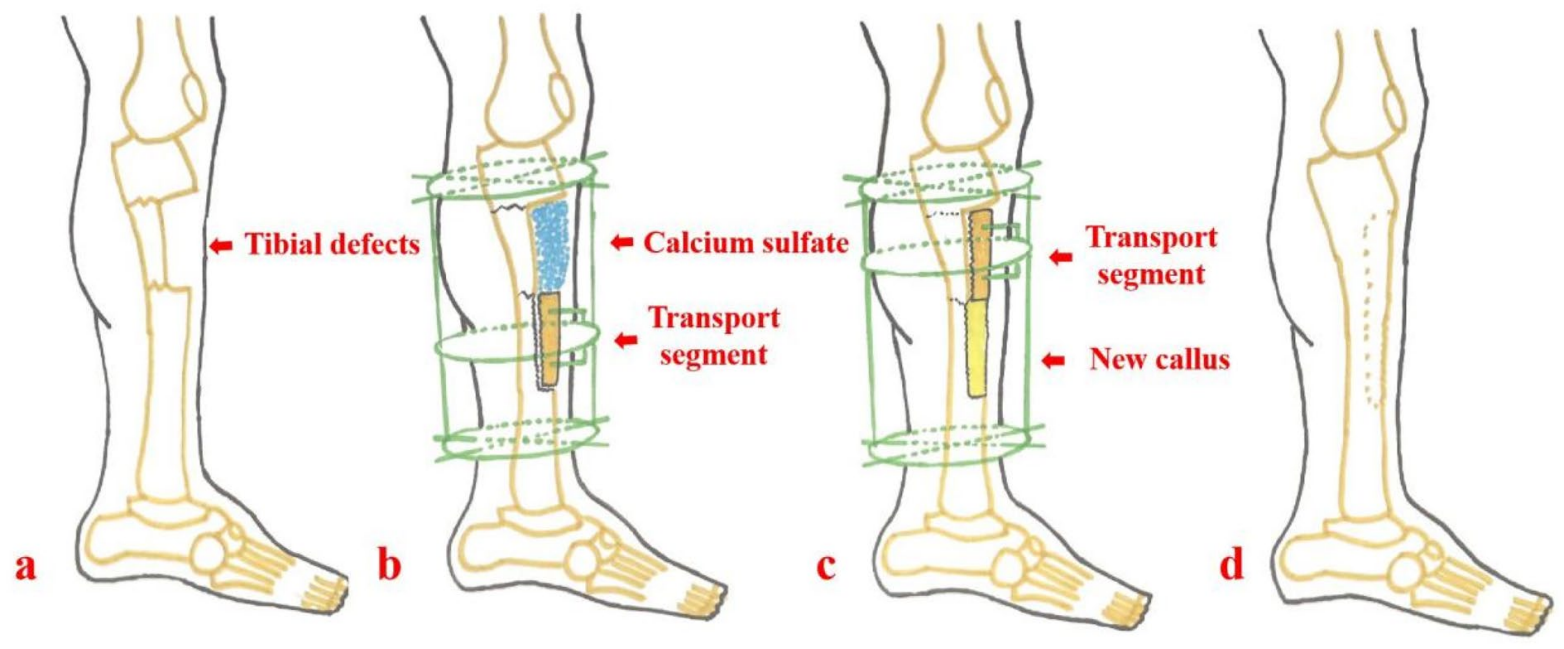
Schematic diagram of the semi-focal bone transport technique. (**a**) The patient suffers from semicylindrical tibial defects; (**b**) The annular frame is installed, semicircular osteotomy is performed and antibiotic-loaded calcium sulfate is filled into the bone defect site; (**c**) New callus generates after gradual semi-focal bone transport; (**d**) After consolidation period finishes and docking site heals, the annular fixator is removed.

Ilizarov bone transport technique can effectively solve the problems of insufficient donor site, nonunion of implanted bones and limb deformity according to the principle of distraction osteogenesis^1,6^. It has been recognized as the golden standard for patients with large bone defects. However, long time wearing an Ilizarov frame leads to lots of frame-related complications, and makes patients feel anxious^8^. In order to shorten the time in frame and reduce the occurrence of complications, we have developed a modified technique of semi-focal bone transport combined with antibiotic-loaded calcium sulfate for patients with large tibial bone defects (as shown in Fig. 1). The first stage was radical debridement and soft tissue reconstruction. During radical debridement, infected and necrotic bones needs to be removed, while healthy bones need to be preserved. The second stage was bone reconstruction using the semi-focal bone transport technique. The bone defect site was filled with antibiotic-loaded calcium sulfate. The purpose of this research was to introduce this technique, and compare it with the traditional Ilizarov bone transport technique.

## Materials and methods

### Study design

The institutional review board (IRB) approval was gotten from the ethics committee of Xi’an Hong Hui hospital. All methods were performed according to relevant guidelines and regulations. This research was conducted using a retrospective analysis design. Sixty-two patients with large tibial bone defects treated in our institution from June 2010 to June 2021 were included. The inclusion criteria were as follows: (1) Patients over 18 years; (2) Patients with tibial bone defects longer than five centimeters; (3) Patients treated by the SFBT or TBT technique; (4) Patients with complete medical records. The exclusion criteria were the following three points: (1) Patients accepted other treatment scheme, not the SFBT or TBT technique; (2) Patients who were unable to tolerate anesthesia or surgery; (3) Patients with incomplete medical records.

The demographic information were gathered via the medical records at the authors’ institution, comprising the age, sex and body mass index (BMI) of the patients. Etiology included acute trauma and osteomyelitis. Involved bone segment was divided into proximal, mid-shaft and distal. Bone defect size and volume were calculated after sequestrum resection using the picture archiving and communication system. The operative time was determined based on the surgical records. The radiographic consolidation for the distraction callus was measured based on the standards of Fischgrund et al.^9^ and Paley et al.^10^ using computed tomography. The bone and functional results were evaluated using the ASAMI scoring system reported by Paley^11,12^. The postoperative complications were recorded and classified as “problems” (treated nonoperatively), “obstacles” (treated operatively) or “sequelae” according to the method used also by Paley^10^. Bone results were evaluated by union, infection, deformity, and limb length discrepancy and classified as excellent, good, fair, and poor. The functional results were assessed based on limp, range of motion of adjacent joints, sympathetic dystrophy, and return to activity, and also classified as excellent, good, fair, and poor.

### Treatment procedure of the two groups

In SFBT group, for a patient suffered from acute trauma or osteomyelitis of the tibia, the first stage was radical debridement, where the goal was infection prevention and eradication. Blood samples were collected to detect erythrocyte sedimentation rate (ESR), C-reactive protein (CRP), white blood cell (WBC) count, etc. Bacterial culture and drug sensitivity test were performed using the wound secretion. Surgical debridement was the focus, including removal of all infected soft tissues, sequestrum and completely free non-vascularized fragments. It could stop until the appearance of good “paprika sign”. Tissues that were difficult to judge vitality could be properly retained and debridement could be carried out again in 48 h. To eliminate dead space, a PMMA spacer loaded with antibiotics was filled into the bone defect site. Then, a temporary external fixator (Tianjin Zhengtian Co.Ltd, China) was inserted to maintain stability. To achieve the most appropriate type of soft tissue reconstruction, the basic “reconstructive ladder” should be followed, including direct suture, skin grafting, local and free flaps.

**Figure 2:**
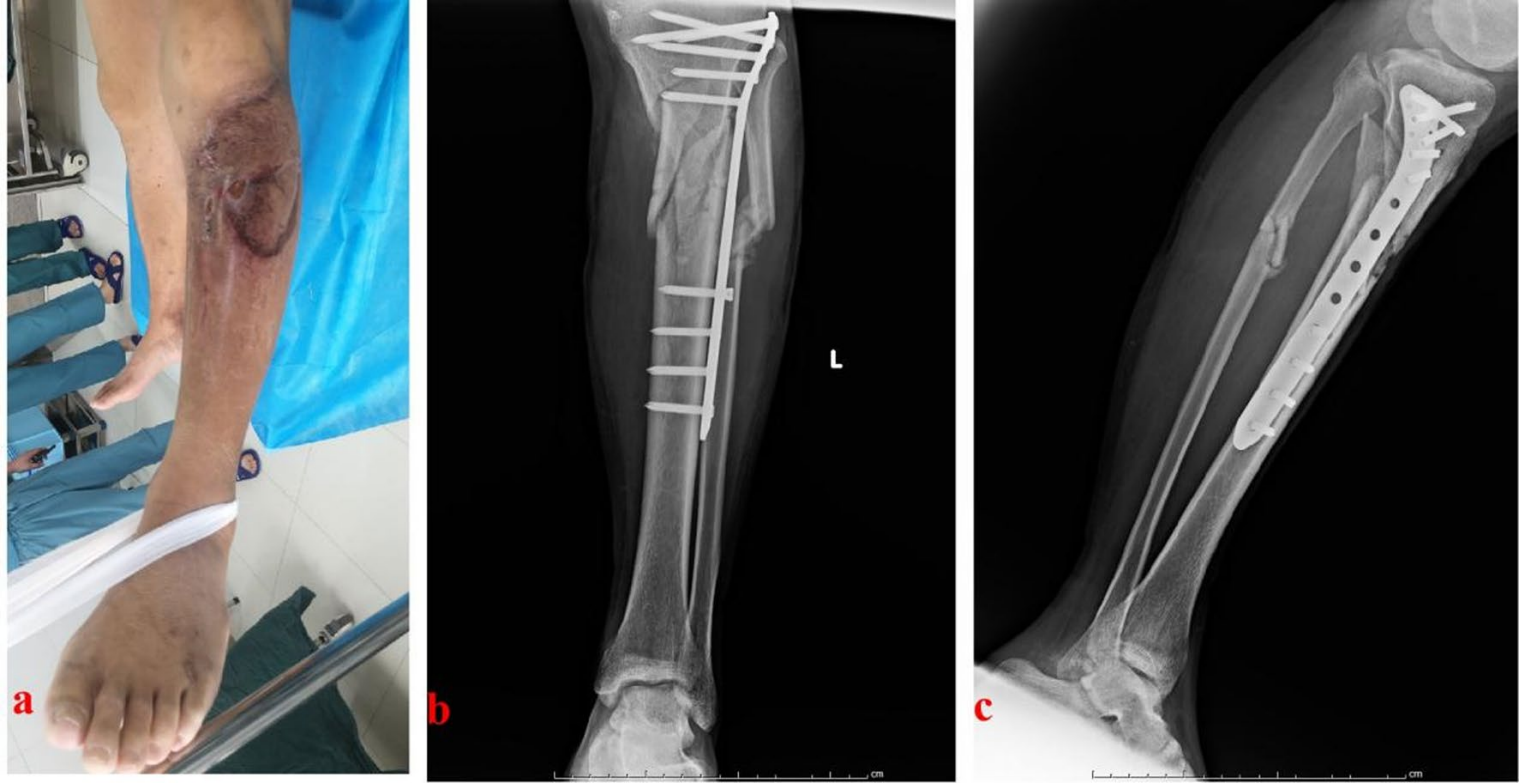
A 43-year-old male suffered from proximal tibial osteomyelitis. (**a**) A sinus could be found from the injured limb; (**b**) and (**c**) The initial X-ray films of the injured lower leg.

**Figure 3:**
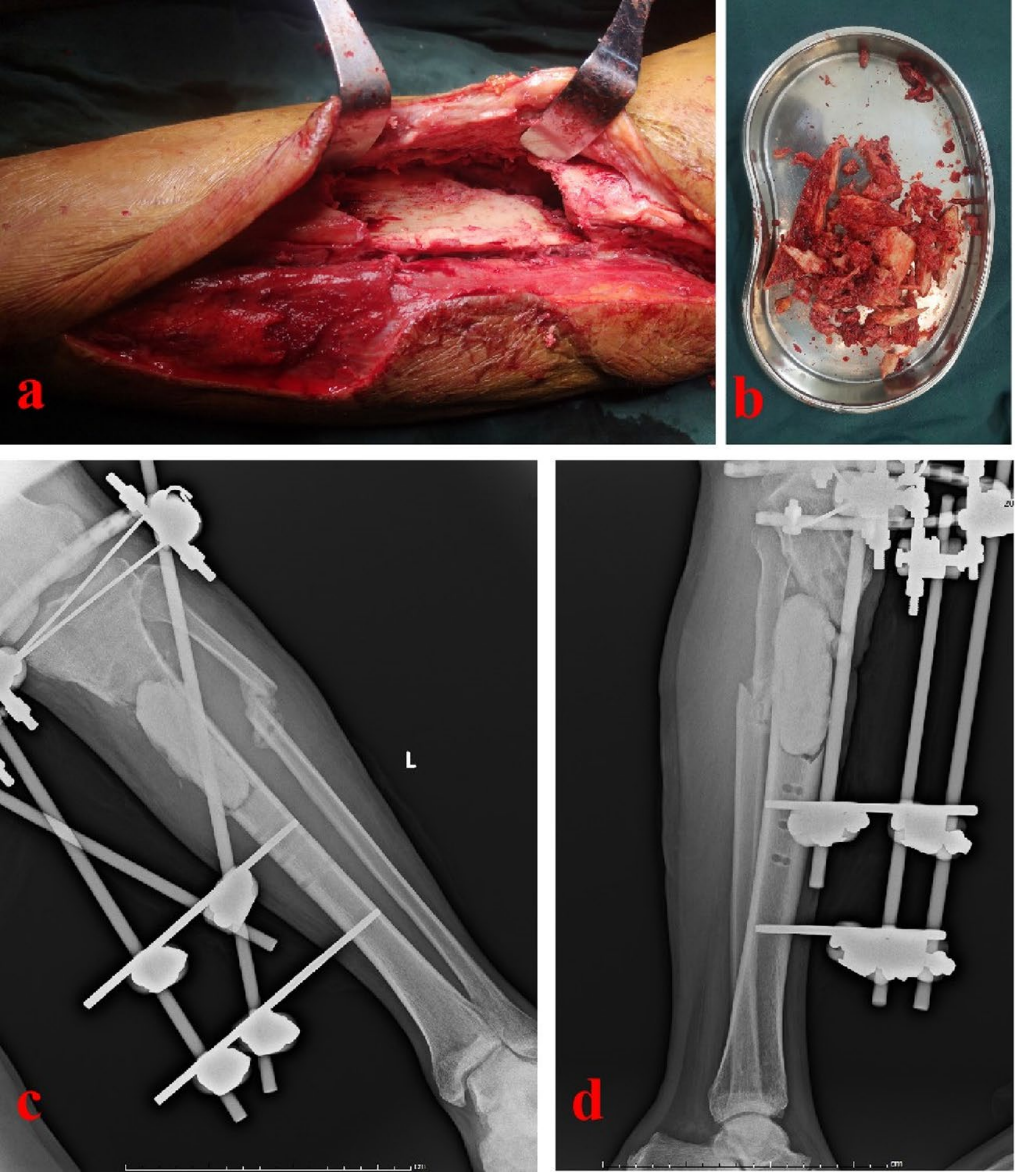
Radical debridement and external fixation were performed for this patient. (**a**) The intraoperative picture showed the status after removal of all sequestrum; (**b**) The tray showed the sequestrum removed during operation; (**c**) and (**d**) X-ray films after debridement showed that, the semicylindrical tibial defect site (about 8.0 cm) was filled with bone cement.

**Figure 4:**
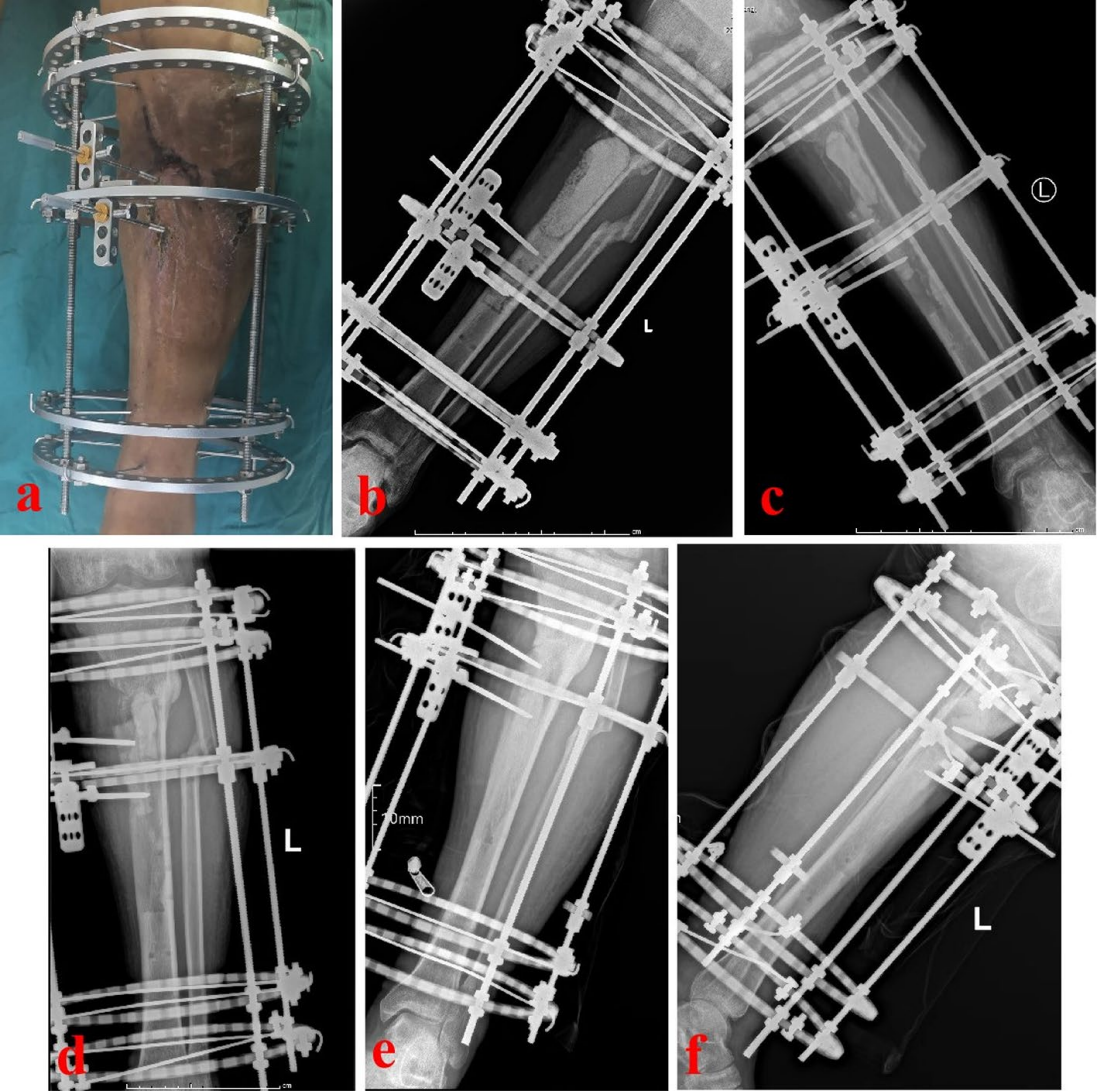
The patient was treated by the semi-focal bone transport technique. (**a**) Appearance of the injured limb during the semi-focal bone transport process; (**b**–**d**) X-ray films of the semi-focal bone transport process; (**e**–**f**) X-ray films showed that the consolidation of new callus was good.

**Figure 5:**
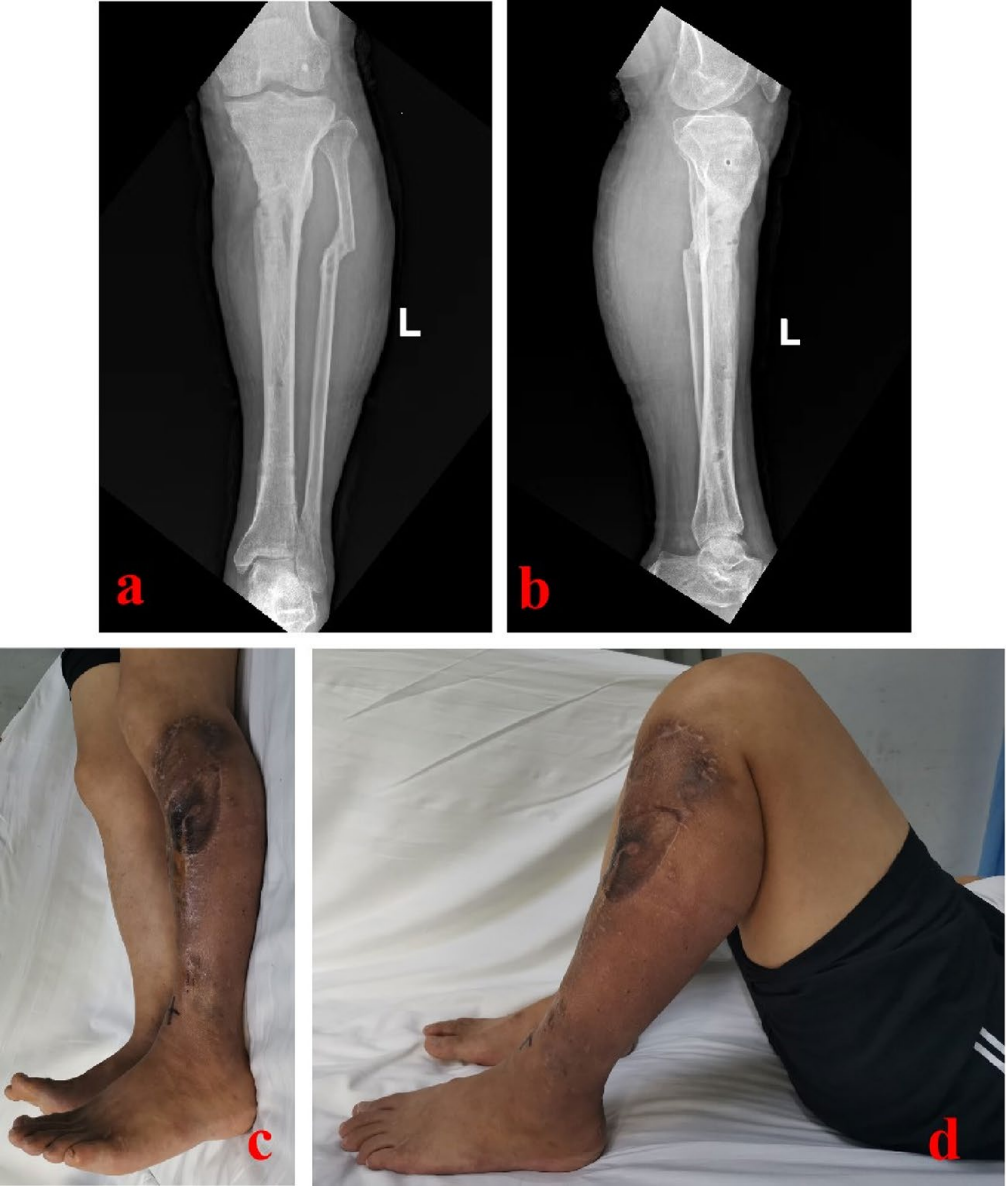
The patient was successfully treated by the modified bone transport technique. (**a**) and (**b**) X-ray films after removal of the annular frame; (**c**) and (**d**) Appearance of the injured limb after removal of the annular frame.

**Figure 6:**
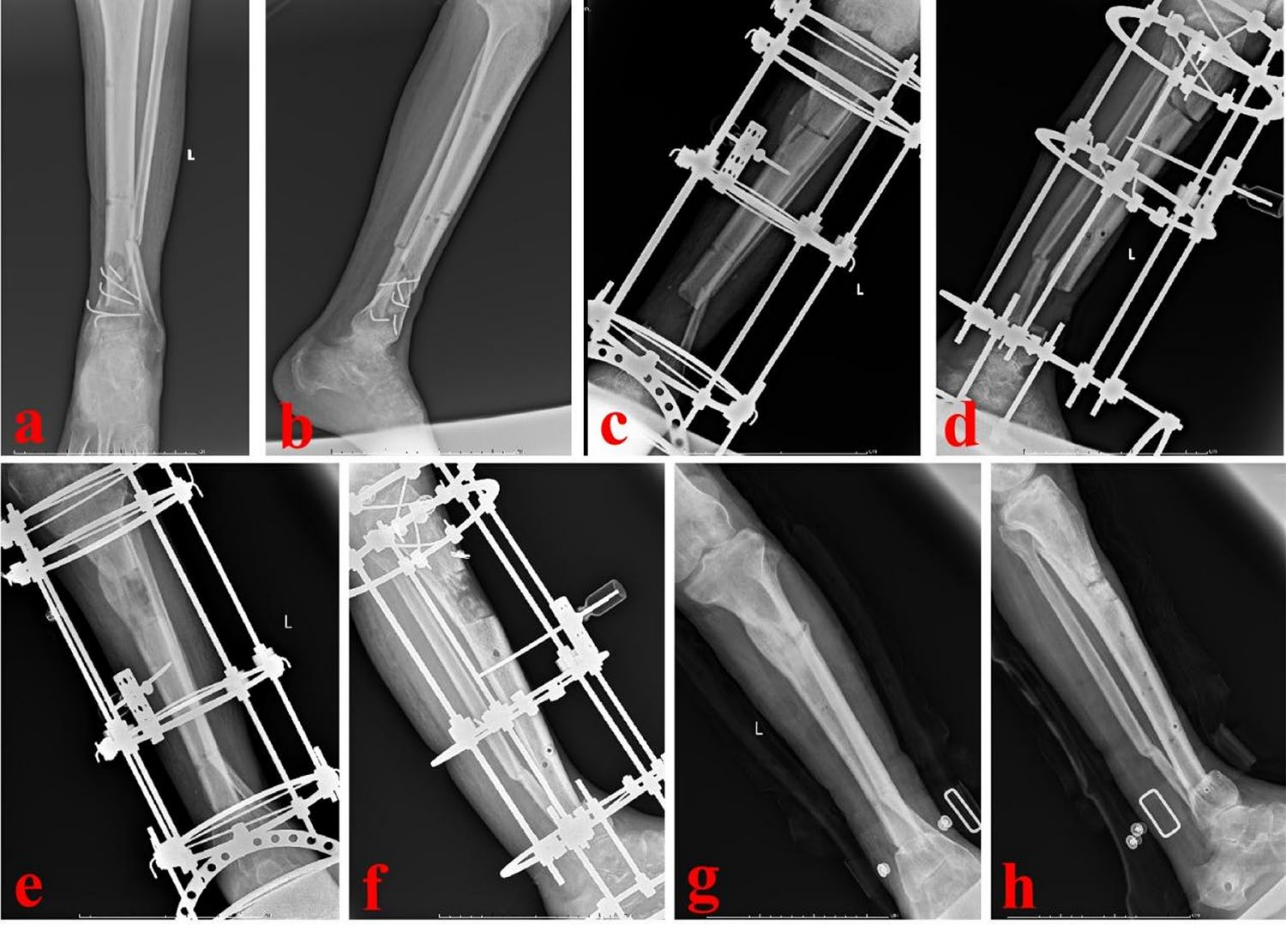
A 56-year-old male was successfully treated by the traditional bone transport technique. (**a**) and (**b**):The patient suffered a severe open fracture and infection; (**c**) and (**d**) After radical debridement, the distal tibial defects occurred. Ilizarov frame was installed and osteotomy was performed; (**e**) and (**f**) Traditional bone transport technique was successfully applied and new callus formed; (**g**) and (**h**) X-ray films after removing the Ilizarov annular frame.

When the infection-monitoring indicators returned to normal and the wounds healed well, the second stage of bone reconstruction was carried out. The temporary external fixator was removed and the PMMA spacer was taken out. The bone defect site was re-examined for signs of infection. The bone defect site was revised into semicylindrical or similar size. The chief surgeon repeatedly determined that there was no active infection locally. Then, the injured leg was maintained in the center of the Ilizarov bone transport frame (Tianjin Zhengtian Co.Ltd, China). Parallel to the knee and ankle joint surface, the Ilizarov fixator was fixed with Kirschner wires. According to the surgical design, semicircular osteotomy was performed. Multiple drill holes technique was used to perform the osteotomy. To avoid thermal damage, a low-speed drill was used. During drilling, physiological saline solution was used for cooling. When performing osteotomy, the chief surgeon was careful not to break the semicylindrical transport segment. Two Schanz nails were inserted to fix the transport segment. Under the C-arm image intensifier, the alignment and rotation were checked. This was to ensure that there was no axial deviation. After that, antibiotic-loaded calcium sulfate spacer was made. Antibiotics were determined based on the preoperative drug sensitivity test. The regular proportion was: calcium sulfate 7.5 g + vancomycin 0.5 g + gentamicin injection 3 ml. With the aid of mixed fluid, the powders were well mixed. Then, the spacer was filled into the semicylindrical or similar bone defect site. The wounds were washed and sutured. One week after operation, the annular frame was operated for semi-focal bone transport. The initial distraction speed was one millimeter per day. The distraction speed was adjusted according to the tolerance of the injured limb. In order to generate appropriate stress stimulation, the injured limb could bear partial weight after the semi-focal bone transport began. When the docking site was in contact, appropriate pressure was applied to promote docking site healing. After the consolidation process finished, the annular frame would be removed. A typical case is shown in Figs. 2, 3, 4, 5. In TBT group, the surgical procedures were the same as those described in previous literature^13^. After debridement, the bone defect site was revised into cylindrical or similar size. A typical case is shown in Fig. 6.

### Postoperative treatment

All patients were given anti-inflammatory, detumescence, pain relief and other symptomatic treatments. Sensitive antibiotics were applied for four to six weeks. According to the drug sensitivity test, antibiotics were adjusted in time. Infection-related indicators were detected regularly. All patients were guided to participate in functional exercises after operation. X-ray images were collected once every 2 weeks. In order to monitor the generation of distraction callus, computed tomography of the injured limb was performed if necessary. Local care was given for all pin-tracts.

### Statistical analysis

Statistical analysis was performed in SPSS software 24.0 (Chicago, IBM Company, United States). Measurement data were expressed as mean ± standard deviation by unpaired *t* test. Count data were analyzed using χ^2^ test. *p* < 0.05 was defined as statistically significant.

## Results

### Demographics

Table 1 shows the basic characteristics of the two groups. The mean age was 37 ± 10 years and 38 ± 12 years for SFBT and TBT patients (*p* > 0.05). There were 18 males and nine females in SFBT group while 21 males and 14 females in TBT group, respectively (*p* > 0.05). The mean BMI of the two groups was 26 ± 4 kg/m^2^ and 25 ± 5 kg/m^2^ (*p* > 0.05). Ten cases suffered from acute trauma and 17 were diagnosed with osteomyelitis initially in SFBT group. In TBT group, there were 16 cases suffering from acute trauma and 19 from osteomyelitis. The mean bone defect size after radical debridement was 7.7 ± 1.6 cm which were located proximal in seven patients, mid-shaft in 12 and distal in eight patients of the SFBT group. For the TBT group, it was 7.5 ± 2.1 cm which were located proximal in nine patients, mid-shaft in 15 and distal in 11 patients. The mean bone defect volume was 20.4 ± 5.6 cm^3^ for SFBT patients and 38.8 ± 7.2 cm^3^ for TBT patients (*p* < 0.001). The average follow-up duration after removal of the fixator was 30 ± 5 months and 32 ± 6 months for SFBT and TBT patients (*p* > 0.05).

**Table 1:** Demographic data of the two groups.

Variable	SFBT group (n = 27)	TBT group (n = 35)	*p* value
Age (years)	37 ± 10	38 ± 12	*p* = 0.722
Sex (M/F)	18/9	21/14	*p* = 0.590
BMI (kg/m^2^)	26 ± 4	25 ± 5	*p* = 0.385
Etiology			*p* = 0.492
Acute trauma	10	16	
Osteomyelitis	17	19	
Involved bone segment			*p* = 0.987
Proximal	7	9	
Mid-shaft	12	15	
Distal	8	11	
Bone defect size (cm)	7.7 ± 1.6	7.5 ± 2.1	*p* = 0.672
Bone defect volume (cm^3^)	20.4 ± 5.6	38.8 ± 7.2	*p* < 0.001
Follow-up (months)	30 ± 5	32 ± 6	*p* = 0.159

BMI stands for body mass index. SFBT stands for semi-focal bone transport. TBT stands for traditional bone transport.

### Clinical evaluation

The mean distraction duration was 3.6 ± 1.1 months for SFBT patients and 4.0 ± 1.2 months for TBT patients (*p* > 0.05). The mean distraction index of the two groups was 0.50 ± 0.15 months/cm and 0.54 ± 0.18 months/cm, respectively (*p* > 0.05). Yet, the mean external fixation index was 1.51 ± 0.14 months/cm for SFBT patients and 1.89 ± 0.25 months/cm for TBT patients, with significant difference between the two groups (*p* < 0.001). The mean operative time was 141 ± 19 min for SFBT patients and 132 ± 24 min for TBT patients (*p* > 0.05). ASAMI score was introduced to evaluate the bone defect healing and limb functions. The bone results were excellent in 10 patients, good in 12 patients and fair in five patients of the SFBT group while it was excellent in 11 cases, good in 15 cases and fair in nine cases of the TBT group (*p* > 0.05). For functional results, it was excellent in nine patients, good in 11 patients, fair in five patients, and poor in two patients of the SFBT group while excellent in eight cases, good in 16 cases, fair in eight cases, and poor in three cases of the TBT group (*p* > 0.05). These data are summarized in Table 2.

**Table 2:** Clinical effects of the two groups.

Variable	SFBT group (n = 27)	TBT group (n = 35)	*p* value
Distraction (months)	3.6 ± 1.1	4.0 ± 1.2	*p* = 0.178
Distraction index (months/cm)	0.50 ± 0.15	0.54 ± 0.18	*p* = 0.344
External fixation index (months/cm)	1.51 ± 0.14	1.89 ± 0.25	*p* < 0.001
Operative time (min)	141 ± 19	132 ± 24	*p* = 0.105
Bone results			*p* = 0.779
Excellent	10	11	
Good	12	15	
Fair	5	9	
Functional results			*p* = 0.835
Excellent	9	8	
Good	11	16	
Fair	5	8	
Poor	2	3	

### Complications

Table 3 displays the complications of the two groups. For SFBT patients, 16 problems, which happened during the research period could be treated non-surgically, including six cases for Grade-II pin-tract infection, six for aseptic exudation and four for transient loss of joint movement. Patients suffered from Grade-II pin-tract infection were given local care and systemic antibiotics. Those with aseptic exudation were managed by dressing change. Patients encountered transient loss of joint movement were instructed to participate in functional exercises or manual release. Eleven obstacles led to revision surgery in SFBT group, which comprised five cases with Grade-III pin-tract infection, one case with infection recurrence, three cases with docking site nonunion, and two cases with axial deviation. The infected pins were removed and a new pin was inserted for patients with Grade-III pin-tract infection. The one suffered from infection recurrence underwent radical debridement again and was given systemic antibiotics. After infection was completely controlled, bone transport continued. Those with docking site nonunion were treated by autocancellous bone graft from iliac crest. Patients encountered axial deviation were managed by adjusting the transport frame. Three patients suffered from sequelae and rejected further corrections in SFBT group.

**Table 3:** Complications.

Variable	SFBT group (n = 27)	TBT group (n = 35)	*p* value
Problems			
Grade-II pin-tract infection	6 (22.2%)	16 (45.7%)	–
Aseptic exudation	6 (22.2%)	–	–
Transient loss of joint movement	4 (14.8%)	7 (20.0%)	–
Obstacles			
Grade-III pin-tract infection	5(18.5%)	11 (31.4%)	–
Infection recurrence	1 (3.7%)	2 (5.7%)	–
Docking site nonunion	3 (11.1%)	9 (25.7%)	–
Axial deviation	2 (7.4%)	7 (20.0%)	–
Sequelae	3 (11.1%)	4 (11.4%)	–
Number of complications per patient	1.1 ± 0.6	1.6 ± 0.7	*p* = 0.004

In TBT group, 23 problems occurred, including 16 patients for Grade-II pin-tract infection and seven cases for transient loss of joint movement. Twenty-nine obstacles happened in TBT group, comprising 11 cases for Grade-III pin-tract infection, two cases for infection recurrence, nine cases for docking site nonunion, and seven cases for axial deviation. Four cases suffered from sequelae. The management of these complications in TBT group was similar to that of SFBT patients. The mean number of complications per patient was 1.1 ± 0.6 for SFBT patients and 1.6 ± 0.7 for TBT patients, and the difference was significant (*p* < 0.05).

## Discussions

The optimal treatment for patients with large tibial bone defects is still controversial. It attracts considerable critical attention which requires individualized elaborate reconstructive strategy to achieve expected effects. Methods of reconstruction have been proposed, including different kinds of bone grafting and Ilizarov bone transport technique. Whether it is direct bone grafting, bone grafting in induced membrane or fibular bone grafting, it is essentially a bone transplantation technique^1–4^. When Ilizarov technique is used, the large tibial bone defects are usually revised into a cylindrical complete defects regularly, so that the traditional bone transport technique can be performed smoothly.

Autologous bone grafting is the first option for the management of bone defects as the bone blocks have good histocompatibility and no immune rejection after transplantation. It also shows perfect bone conduction, induction and osteogenesis vitality. However, the size and amount of autologous bones are limited, and the amount varies wildly due to the individual differences of each patient^14,15^. For segmental bone defects that are longer than five centimeters, there will be uncontrollable bone necrosis and resorption. Therefore, the curative effects of non vascularized autologous bone grafting are not accurate^16,17^. Bone grafting in the induced membrane can deal with larger bone defects and reduce the occurrence of bone resorption. Patients achieved success approximately 80 to 82% by using induced membrane technique on the first treatment course^18,19^. After additional surgeries the success rate was about 90%. This technique can cure large tibial bone defects up to 25 cm in length, and limb functions can be recovered after several months. Debnar et al. retrospectively analyzed ten patients using induced membrane technique. Observed complications included two re-fractures, two cases of infection recurrence and one case for delayed healing^20^. Fung et al. found that patients with tibial defects, and those with larger defects, were at significantly higher odds of developing a postoperative infection^21^. Once the deep infection relapses, the results are often disastrous. The pre implanted autologous bones may need to be completely removed. Even if the infection is controlled again, it may not be possible to repair the bone defects by membrane bone grafting. Hsu et al. showed that initial infected nonunion and defect length greater than seven centimeters were risk factors for post-infection of induced membrane technique^22^. Yet, using Ilizarov bone transport will overcome the above disadvantages.

Ilizarov bone transport technique has been considered the golden standard for patients with large segmental tibial bone defects, which could play multiple roles at the same time, including eradicating infection, reconstruction of bone and soft tissue defects, deformity correction, etc. However, long time wearing an Ilizarov frame leads to lots of frame-related complications, and makes patients feel anxious^8^. Besides, traditional bone transport technique is usually used to repair a complete cylindrical bone defect. During debridement, segmental resection is often used to completely remove the infected bone tissues. Although this debridement method is radical, sometimes too much normal bone tissues will inevitably be removed. This may prolong the bone transport time and lead to the occurrence of frame-related complications. Incomplete bone defects after debridement are more common in clinical work, especially some semicylindrical or similar bone defects. For example, in patients with infection after plate internal fixation, the infected bones under the plate often need to be removed, while the bones on the other side can usually be retained.

Based on these factors, we developed a modified technique of the semi-focal bone transport combined with antibiotic-loaded calcium sulfate. Several studies of traditional bone transport technique have shown that the external fixation index ranged from 1.2 to 2.8 months/cm with a mean of 2.0 months/cm^1,6,23,24^. Our results showed that the mean external fixation index of SFBT technique was significantly lower than that of the TBT technique. The reason may be that when using SFBT technique, the reserved semi-cylindrical bones on the other side could heal early, allowing for early removal of the transport frame. In addition, the excellent and good rate was 81.5% for bone results and 74.1% for functional results using SFBT technique. These results were similar to that of using TBT technique. This means that using SFBT technique could also achieve good bone healing and limb functions.

The mean number of complications per patient using SFBT technique was significantly lower than that of using TBT technique. This may be because patients in the SFBT group experienced a shorter time wearing the transport frame. Therefore, there were fewer frame-related complications in SFBT group. Most of the complications encountered in SFBT group were pin-tract infection (40.7%) and aseptic exudation (22.2%). Aseptic exudation is caused by the absorption of calcium sulfate. Calcium sulfate itself has good biocompatibility and degradability^25^. In other studies, the aseptic exudation incidence after calcium sulfate application was 4–51%^26,27^. We observed that aseptic exudation usually did not occur when the local soft tissue conditions of the tibia were good. On the contrary, if the soft tissues were seriously damaged, it was easy to produce aseptic exudation, which could last for several weeks to several months. Generally, only local care is required for the management of aseptic exudation. The incidence of other complications was low in SFBT group, such as docking site nonunion (11.1%), infection recurrence (3.7%) and axial deviation (7.4%). This may be because antibiotic-loaded calcium sulfate has good occupying effects, so as to prevent soft tissue embedding. Without soft tissue embedding, the docking site will heal more easily. In addition, it can release antibiotics gradually and play an anti-inflammatory role. Semicylindrical or similar bone defects mean that the other half without local defects can be contacted and healed early. This can provide initial stability in local areas and reduce the occurrence of axial deviation and docking site nonunion. This is also conducive to early functional exercise, so as to improve functions of the injured limb.

This research has several limitations, including small sample size and retrospective design. A further prospective research is needed in the future to address methodological limitations. Besides, the chief surgeon may have personal preferences when choosing treatment plans, which may lead to certain biases in this study. Moreover, in cases of semi-cylindrical bone defects, the induced membrane technique might be sufficiently effective. At this point, it is still unclear whether using SFBT technique is superior to induced membrane technique. We will study this issue in further research. Yet, these limitations will not prevent us from introducing our experience of this modified technique.

## Conclusion

Patients with large tibial bone defects were successfully treated by the semi-focal bone transport technique combined with antibiotic-loaded calcium sulfate or the traditional bone transport technique. Compared to the traditional bone transport technique, patients using the semi-focal bone transport technique achieved better clinical effects, including shorter EFI and less complications. This modified technique could be one of the treatment options for such patients.

## Data Availability

The datasets analyzed during the current study are available from the corresponding author upon reasonable request.
